# Nrf2: a main responsive element in cells to mycotoxin-induced toxicity

**DOI:** 10.1007/s00204-021-02995-4

**Published:** 2021-02-08

**Authors:** Marta Justyna Kozieł, Karolina Kowalska, Agnieszka Wanda Piastowska-Ciesielska

**Affiliations:** Medical University of Lodz, Department of Cell Cultures and Genomic Analysis, Zeligowskiego 7/9, 90-752 Lodz, Poland

**Keywords:** Mycotoxins, Nrf2, Oxidative stress, Antioxidants

## Abstract

Nuclear factor erythroid 2-like 2 (Nrf2) is a transcription factor participating in response to cellular oxidative stress to maintain the redox balance. Generation of reactive oxygen species (ROS) and, in consequence, oxidative stress, are physiological as well as pathological processes which take place in almost all types of cells. Nrf2, in response to oxidative stress, activates expression and production of antioxidant enzymes to remove free radicals. However, the role of Nrf2 seems to be more sophisticated and its increased expression observed in cancer cells allows to draw a conclusion that its role is tissue—and condition—dependent. Interestingly, Nrf2 might also play a crucial role in response to environmental factors like mycotoxins. Thus, the aim of the study is to review the role of Nrf2 in cells exposed to most common mycotoxins to check if the Nrf2 signaling pathway serves as the main response element to mycotoxin-induced oxidative stress in human and animal cells and if it can be a target of detoxifying agents.

## Introduction

Exposure of human and animals to environmental pollutants: drugs, food additives, pesticides, toxins, ionizing and ultraviolet radiation contributes to formation of high-energy molecules: reactive oxygen species (ROS) and, in consequence, to oxidative stress. Physiological levels of ROS are necessary in cells to assure normal proliferation and growth, signal transduction as well as activation of antioxidants and anti-apoptotic signals (Banerjee et al. 2020). On the other hand, high levels of ROS lead to disturbed homeostasis and may damage cellular components (Rahal et al. 2014). Thus, a balance between ROS levels and activity of detoxifying enzymes is crucial in maintaining health. Cells are able to defend themselves against stressful conditions using a variety of mechanism. The basic one is synthesis and activation of antioxidant enzymes. The nuclear erythroid 2-related factor (Nrf2) plays a significant role in response to oxidative stress (Enomoto et al. 2001; McMahon et al. 2001; Aoki et al. 2001). It was observed that Nrf2-knockout mice (*Nrf2*^−/−^) are more sensitive to ROS than wild type mice (Loboda et al. 2017b). The *Nrf2* gene (*Nfe2l2*) in humans is located in locus 2q31.2. The protein product of this gene belongs to the cap‘n’collar subfamily and its mass is about 60 kDa. According to the UniProtKB database, this gene is expressed in various tissues and its highest expression is observed in muscle, kidney, lung and liver. *Nfe2l2* may be regulated on different levels: transcriptional, post-transcriptional and via epigenetic modifications. At the transcription level, Nrf2 expression is regulated by the aryl hydrocarbon receptor (AhR), nuclear factor kappa-light-chain-enhancer of activated B cells (NF-κB) and by the *Nrf2* gene itself (Kwak et al. 2002; Miao et al. 2005; Rushworth et al. 2012). It is believed that Nrf2 expression is also regulated at the epigenetic level (DNA methylation, histone modifications, miRNA) (Guo et al. 2015; Cheng et al. 2016) but this process seems to be very complex and needs to be more elucidated.

**Fig. 1:**
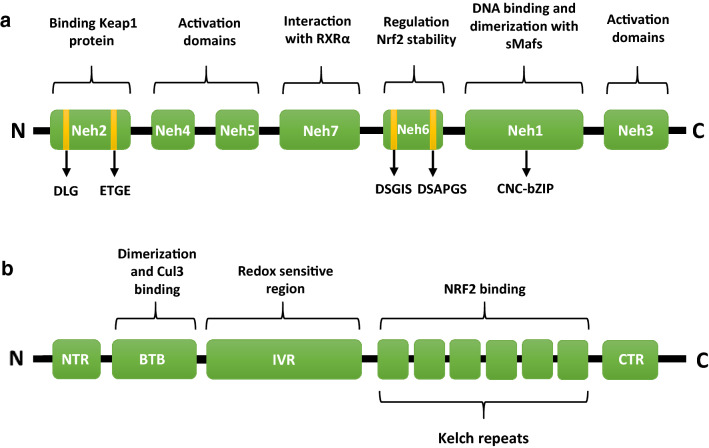
Structure of the Nrf2 protein (**a**) and Keap1 protein (**b**). *Keap1* Kelch-like ECH associating protein 1, *RXRα* retinoid X receptor, *sMaf* small musculoaponeurotic fibrosarcoma proteins, *CNC-bZIP* Cap‘n’Collar-basic leucine zipper region, *Cul3* Cullin 3, *N* N terminal end, *C* C terminal end

Nrf2 consists of seven homology domains (Neh1-7), called Nrf2-ECH (Neh) domains (Hayes and Dinkova-Kostova 2014) (Fig. 1). All of them play an important role in the function of Nrf2: the Neh1 domain contains the Cap‘n’Collar (CNC)-bZIP region which is responsible for dimerization with small Maf (sMAF) proteins and DNA binding (Hirotsu et al. 2012). sMAF proteins heterodimerized by Nrf2 are: MafF, MafG and MafK (Motohashi et al. 2002). The Neh2 domain contains two motifs: DLG and ETGE through which it controls interaction with Keap1 protein (Tong et al. 2006). The Neh3 region is considered responsible for recruitment of chromo-ATPase/helicase DNA-binding protein (CHD) 6 (Hayes and Dinkova-Kostova 2014). The Neh4 and Neh5 regions recruit cAMP response element-binding protein (CREB)-binding protein (CBP) and interact with RAC3 (receptor-associated coactivator 3) (Kim et al. 2013). The Neh6 region contains two peptide motifs: DSGIS and DSAPGS, which are necessary to interact with TrCP (β-transducin repeat-containing protein). After phosphorylation of the DSGIS motif by glycogen synthaze kinase-3β (Gsk-3β), TrCP binds more efficiently to this domain and promotes proteasomal degradation of Nrf2 via Skp1-Cul1-F-box (SCF) ubiquitin ligase complex (Tonelli et al. 2018). And the last domain of Nrf2, i.e., Neh7 interacts with the retinoid X receptor (RXRα) and thus mediates the repression of transcriptional activity of Nrf2 (Wang et al. 2013). Interestingly, regulation of the Nrf2 transcriptional factor may have therapeutic applications in many diseases. However, it is worth emphasizing that a lot of these activators have a toxic effect on the human body (Abed et al. 2015; Kerr et al. 2017).

**Fig. 2:**
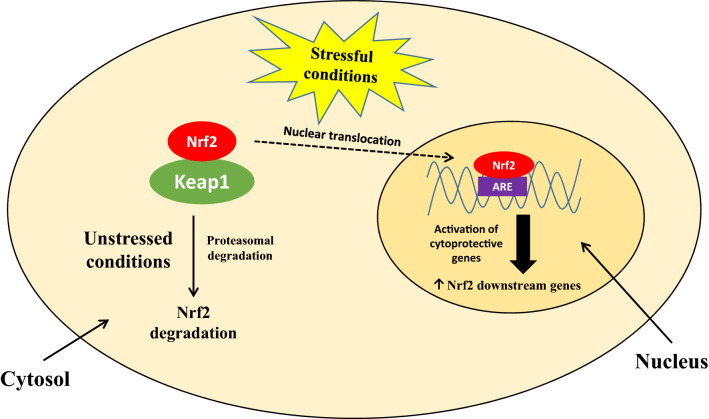
Schematic diagram showing the behaviour/activity of Nrf2 under stressful and homeostatic conditions. *ARE* antioxidant responsive element

In homeostatic conditions, Nrf2 occurs in cellular cytoplasm and is connected with Keap1 (Kelch-like ECH associating protein 1); in stressful conditions, Nrf2 dissociates from the inactive Keap1-Nrf2 complex and translocates to the nucleus, where it regulates the expression of genes associated with detoxification (Fig. 2). Multiple mechanisms are involved in dissociation of Nrf2 from Keap1. Some of them might affect Keap1-main regulator, while others can directly interact with Nrf2. Mechanisms that affect the Keap1 protein include: oxidation of cysteine residues in Keap1, an interaction of p62 with Keap1 and phosphorylation of Nrf2 by Protein kinase C (PKC). Among Keap1-independent mechanisms, β-TrCP-Cul1-Dependent Pathways and Hrf1-Dependent Pathway (Li et al. 2019a) might be distinguished.

The role of Nrf2 in cells is twofold: on the one hand, it protects cells against ROS and decreases DNA damage caused by it, but on the other hand, prolonged expression of this factor is associated with a more aggressive and resistant type of cancers and may be oncogenic (Sporn and Liby 2012). Thus, it seems necessary to evaluate the adverse effect of potential Nrf2 activators in therapy and optimize the dose and exposure to it. Electrophilic and non-electrophilic compounds are two major types of Nrf2 protein activators. Electrophilic inducers interact with cysteine residues of Keap1 and thus inactivate E3 ligase. Non-electrophilic inducers disturb the interaction between the Keap1-Nrf2 complex and in consequence, deactivate the Keap1 E3 ligase activity (Suzuki and Yamamoto 2015). Based on the two-fold role in physiological and pathophysiological processes, a few pharmacological activators as well as a few clinical trials with the studied Nrf2 inhibitors stating Nrf2 signalling pathway as a potential therapeutic target in the future (Al-Sawaf et al. 2015).

Recently, a lot of toxicological studies on mycotoxins and their effect on human health have suggested that activation of Nrf2 or blockage of this pathway might serve as a potential molecular mechanism of observed toxicity of mycotoxins in human and animal species (Loboda et al. 2017b; Yoon et al. 2019; Huang et al. 2020). Thus, this study was to show if mycotoxins act as activators or inhibitors of the Nrf2 signalling pathway and if Nrf2 serves as the main response element to mycotoxins in human and animal cells. We also tried to find out if natural antioxidants modulating the Nrf2 signalling pathway serve as detoxifying agents in mycotoxins exposure in humans and animals.

## Mycotoxins

Mycotoxins are secondary metabolites of fungi which demonstrate a toxic effect on animal and human health. The direct reason why mycotoxins are produced by fungi has not been explained as yet. They are mainly produced by *Aspergillus*, *Fusarium*, *Penicillium* and *Alternaria* species. Mycotoxins can be categorized into four major classes, including: aflatoxins (AFs), ochratoxins (OTA), patulin (PAT) and finally, *Fusarium* toxins, being the most diverse group, including: trichothecenes [deoxynivalenol (DON) and its derivatives, nivalenol (NIV), T-2 and HT-2 toxin], feminises (FMs), zearalenone (ZEA) and its derivatives. Contamination of food with fungi and, consequently, mycotoxins, is contributed by various factors such as: improper harvesting, storage, processing and/or transport of crops. Farm animals present a different degree of sensitivity to mycotoxins; among them, pigs and poultry are reported to be the most sensitive (Yang et al. 2020). The contamination with mycotoxins affects all parts of the food chain, beginning with cultivation, harvesting, processing of food and accumulation in animal tissue. For example, aflatoxin M, a metabolite of aflatoxin B1 may accumulate in eggs (Trucksess et al. 1983; Wolzak et al. 1985) or milk (Nakajima et al. 2004) and be present in the food chain of many products, both processed as well unprocessed. Due to resistance of mycotoxins to heat and chemical treatment during food processing, it is difficult to prevent them from getting contaminated. What is more, mycotoxins occur naturally, thus it is impossible to eliminate them entirely. However, understanding how mycotoxins contamination affects animals and humans is crucial as it enables to identify a proper regulation or possible modulators of toxicity. In response to reports on strong adverse effects of mycotoxins on both humans and animals, the Joint FAO/WHO Expert Committee on Food Additives (JECFA) created the maximum recommended daily intake (Table 1). Also the European Food Safety Authority (EFSA) monitors studies on mycotoxins to determine their tolerable daily doses (De Ruyck et al. 2020). Not only is assessment of concentration of mycotoxins in food samples crucial for mycotoxin studies. Understanding of the toxicity mechanism as well as cellular response to mycotoxin exposure allows to evaluate real effects of mycotoxins on human health.

**Table 1:** Summary information about functions of various mycotoxin and total daily intake doses

Mycotoxin	Effect on body	Total recommended tolerable daily intake (µg/kg)	References
Deoxynivalenol	Genotoxic, impairs the immune response, reduces fertility and embryotoxicity	1.0	Mishra et al. (2014); Yu et al. (2017a); Urbanek et al. (2018); Habrowska-Górczyńska et al. (2019)
Nivalenol	Impairs the immune response, genotoxic, retards growth, cause of reproductive disorders, heameatotoxic and myelotoxic	1.2	Bony et al. (2007); Kongkapan et al. (2016); Schwartz-Zimmermann et al. (2019)
Zearalenone	Genotoxic, teratogenic, immunotoxic and cause of reproductive disorders	0.5	Hueza et al. (2014); Lai et al. (2015); Yang et al. (2018); Zhang et al. (2018a)
Aflatoxins	Mutagenic, carcinogenic, hepatotoxic, teratogenic, immunotoxic, genotoxic	0.00015	Peters and Teel (2003); Stettler and Sengstag (2001); Wangikar et al. (2005); Abnet (2007); Meissonnier et al. (2008); Abdel-Wahhab et al. (2010); Zhang et al. (2016)
Ochratoxin A	Nephrotoxic, mutagenic, carcinogenic, teratogenic, immunosuppressive	0.014	Kumar et al. (2003); Huff et al. (1975); Gupta et al. (2005); Marin-Kuan et al. (2006); Solcan et al. (2013); Malir et al. (2013); Limonciel and Jennings (2014); Chen et al. (2016)
T-2 + HT-2	Immunosuppressive/immunostimulatory^a^	0.06	Wu et al. (2017)

^a^Dependent on dose- high immunosuppressive, low- immunostimulatory

### Trichothecenes

Trichothecenes are a large and structurally diverse group of mycotoxins with average molecular mass between 200 and 500 Da. This group includes a number of mycotoxins. However, it seems that types A and B are most toxic for humans and animals. Generally, their cellular effect is based on the interaction with peptidyltransferase enzyme and binding to the 60S ribosomal subunit, which in consequence, inhibits protein synthesis (Marin et al. 2013). Moreover, trichothecenes affect mitochondrial protein synthesis and are able to interact with protein sulfhydryl group (protein-SH groups)- one of the most potent and ubiquitous ligands in biological systems (McCormick et al. 2011).

T-2 toxin is one of the most dangerous secondary metabolite which belongs to type A of trichothecenes. It is produced by various types of *Fusarium* species. T-2 toxin is metabolized to HT-2 toxin, so it is believed that both the toxins act similarly (Schuhmacher-Wolz et al. 2017). Both mainly occur in cereals (Anfossi et al. 2016). T-2 toxin induces apoptosis and mitochondrial structural disorganization, increases the amount of ROS, and leads to DNA damage (Deyu et al. 2018). Lipophilicity of T-2 is an example of quick absorption by skin, gut and pulmonary mucosa (Zhang et al. 2018b). Neurotoxicity of T-2 was also proven. The effect of T-2 toxin on Nrf2 expression may be different and depend on the time and dose. As Deyu et al. observed in their study, short exposure to T-2 toxin administered in low in doses, resulted in an increased expression of PKA signaling pathway, which in turn led to an increased expression of the Nrf2 transcription factor and PTEN-induced protein kinase 1 (PINK1) (Deyu et al. 2018). This observation is confirmed by the fact that GH3 cells with knock- outed *Nrf2* and exposed to T-2 toxin presented a decreased expression of PINK1, which in consequence confirmed that PKA/Nrf2/PINK1 signaling pathway is involved in T-2 toxicity in GH3 cells (Deyu et al. 2018). Moreover, in higher doses and during longer exposure, the expression of the Nrf2 transcription factor was downregulated (Chaudhary and Lakshmana Rao 2010; Zhang et al. 2018b). Inhibition of Nrf2 was associated with oxidative stress, mitochondrial dysfunction and activation of p53 in mouse neuroblastoma 2a (N2a) cells (Zhang et al. 2018b). Nrf2 expression was also significantly decreased in mice exposed to T-2 at a dose of 5.94 mg/kg as well as phase II detoxifying enzymes NQO1, GCLM and HO-1 (Chaudhary and Lakshmana Rao 2010). This fact suggests that a higher dose of T-2 toxin impairs cellular response to oxidative stress and hereby leading to disruption of the protective pathways. However, it seems necessary to elucidate this concept and more research studies are needed to understand the molecular mechanism of T-2 toxin. Nevertheless, all studies confirmed that the Nrf2 pathways is attributed to cellular oxidative stress caused by T-2.

Deoxynivalenol (vomitoxin; DON) belongs to type B of the trichothecenes family of mycotoxin produced mainly by *Fusarium* species (Urbanek et al. 2018) and is considered one of the best known mycotoxins. It is detected in 90% of tested samples (Sobrova et al. 2010). DON is considered to be one of the most dangerous pollutants occurring naturally in cereal grains, due to its high resistance to processing, grinding and heating (Sugita-Konishi et al. 2006) and is reported to trigger apoptosis, immune response and oxidative stress in cells (Mishra et al. 2014) via disruption of the normal function of mitochondria. DON affects various signaling pathways: RNA-activated protein kinase R (PKR) and hematopoietic cell kinase (Hck), Mitogen-Activated Protein Kinases (MAPKs) (Bae and Pestka 2008). In addition, DON also affects ERK, p38 and JNK and its effect is time- and dose- dependent (Zhou et al. 2003). The main cellular effect of DON includes ribosomal stress in cells—binding of DON to the 28S ribosomal RNA peptidyltransferase site, inhibition of protein synthesis—a process called ribotoxic stress response, characteristic for all this group of mycotoxins. Toxic exposure to DON is manifested by vomiting, nausea and diarrhea (Pinton and Oswald 2014).

Mishra et al. observed that the effect of DON on Nrf2 expression was twofold: after 6 and 12 hours of exposure, the expression of Nrf2 got increased, but after 24 hours it decreased (Mishra et al. 2016). Activation of the ERK1/2 signaling pathway might have implied that DON inhibits translocation of Nrf2 to the nucleus in human HaCat keratinocytes and thus inhibits transcription of genes involved in the defense mechanism during oxidative stress. The same authors concluded that DON might participate in skin carcinogenesis (Mishra et al. 2016), in which the role of Nrf2 is also controversial (Gęgotek and Skrzydlewska 2015). Intestinal epithelial cells (IECs) are exposed to different factors and constitute the first line in contact with mycotoxins Thus, it is necessary to identify the effect of mycotoxins on intestinal cells to assess a real effect of mycotoxins on human health. In the human colon, adenocarcinoma cell line HT-29 DON disrupted the transport of various components and thus led to disturbance in cellular homeostasis (Maresca et al. 2002). DON-contaminated animal feed decreases nutrient absorption, and in consequence triggers low mass of pigs, mainly in consequence of oxidative stress generated in intestinal cells and activation of Nrf2 and NFκB signaling pathways (Zha et al. 2020a). Disruption of intestinal cells caused by DON as well as activation of the Nrf2 signaling pathway and modulation of antioxidants levels were also observed in juvenile grass carp (Huang et al. 2018). A similar effect was observed in human leukemic T cell lymphoblast Jurkat-1 cells with activation of NFκB, MAPK and endoplasmic reticulum (ER)-stress induced apoptosis (Katika et al. 2015) indicating that these signaling pathways are the main response elements in different cell lines to DON exposure.

Del Regno et al. presented that nivalenol (NIV), which is another mycotoxin belonging to the trichothecenes group, may cause oxidative stress in human cells. Moreover, it can enhance the effect of DON (Del Regno et al. 2015; Alassane-Kpembi et al. 2017). The cross-contamination with different mycotoxins is commonly observed in food samples. The same research group revealed that in response to oxidative stress induced by NIV or/and DON (mostly when they occur together), the Nrf2 and NF-kB pathways are activated, which suggest that cells defend themselves in this way against damage induced by these mycotoxins (Del Regno et al. 2015). These results are consistent with later reports (Adesso et al. 2017), where the expression of HO-1 is increased during induction, which might be directly associated with activation of Nrf2. This study also showed that NIV and/or DON induces oxidative stress and inflammation in intestinal cells and, in consequence, might participate in common intestinal inflammatory diseases (Del Regno et al. 2015). Another study revealed that single exposure of DON in common carp causes oxidative stress and activates the Nrf2 signaling pathway, explaining liver damage being an effect of exposure to mycotoxins (Kövesi et al. 2020a).

Activation of Nrf2 was also observed in placenta during pregnancy. Yu et al. confirmed that DON induces accumulation of ROS in the placenta and thus leads to structural and functional damage, which negatively affects pregnancy (Yu et al. 2017b, 2018b). Moreover, DON through oxidative stress causes disorders in the embryo’s skeleton: cranial/cervical, rib, clavicle, axial skeleton and limb deformations (Yu et al. 2017b). The Nrf2/HO-1 pathway also appeared to be activated to protect placenta and foetus against oxidative stress (Yu et al. 2017b). Thus, the Nrf2/HO-1 pathway is activated in response to oxidative stress induced by DON and seems to be responsible for DON-caused embryotoxicity. This hypothesis seems to be confirmed by subsequent studies in which DON causes maternal liver damage during pregnancy. However, increased expression of Nrf2/HO-1 reduces the amount of reactive oxygen species and thus protect hepatocytes against toxicity of this mycotoxin (Yu et al. 2019). Taken together, although ribotoxic stress is believed to be the main cellular response of DON in cells, this literature study allows to conclude that oxidative stress and activation of its main response element, i.e., the Nrf2 signaling pathway, is always associated with the influence of DON on different cells and different individuals.

### Zearalenone

Zearalenone (ZEA, F-2) is a mycotoxin produced mainly by *Fusarium* species. ZEA is a xenoestrogen due to its estrogenic activity caused by chemical similarity to naturally occurring estrogens, mainly 17β-estradiol that is why, it is called mycoestrogen. ZEA is able to bind to estrogen receptors [mainly estrogen receptor α (ERα) and/or estrogen receptor β (ERβ)], disturbs hormonal homeostasis and thus leads to disturbed functioning of the reproductive system, both in humans and animals (Kowalska et al. 2016, 2018). In consequence, it may lead to the development and progression of hormone-dependent cancer such as breast, cervical, endometrial, ovarian and prostate cancer (Ahamed et al. 2001; Mungenast and Thalhammer 2014; Bronowicka-Kłys et al. 2016). Exposure to mycoestrogens is associated with many different ways of providing: food, water, pesticides, air, cosmetics, plants and medicines. Contamination with ZEA occurs mostly in corn, wheat, rice and barley. Living organisms are able to defend itself against xenobiotics in many different ways, one of which is the phase I and II detoxication enzymes produced in the liver. However, based on research reports the phase I metabolites of ZEA α-zearalenol (α-ZOL) and β-zearalenol (β-ZOL) are also estrogenic and even more estrogenic that ZEA itself (Catteuw et al. 2019). On molecular level ZEA both stimulates the proliferation of cells in low doses as well as induces apoptosis, autophagy and oxidative stress in higher doses (Zheng et al. 2018). Although there is a lot of studies concerning the effect of ZEA on human and animal cell lines, only a few of them evaluated the activation of Nrf2 by ZEA. PI3K-Akt, Nrf2 and ER-stress were associated with the effect of ZEA on murine Leydig cells and associated with apoptosis and oxidative stress. Moreover, the same study confirmed that curcumin might protects Leydig cells from ZEA-induced oxidative stress via reduction of total expression and increase in nucleus expression of Nrf2 (Chen et al. 2020). Similar study was conducted on mice Sertoli cells, where ZEA induced apoptosis via Nrf2/ARE signalling pathway associated with oxidative stress induction in cells (Long et al. 2017). Yoon et al. showed that ZEA led to increased expression of Nrf2 in human liver cancer cells lines (HepG2); moreover, this relationship was only observed in smaller concentration of this mycotoxin for which usually the estrogenic pro-proliferative effect is observed. Oxidative damage caused by ZEA was also observed in mice liver, where ZEA significantly reduced expression of Nrf2 (Long et al. 2016). An increased expression of Nrf2 was associated with apoptosis, ER-stress and autophagy in human liver cells (Yoon et al. 2019). Similar relationship was observed in another study: Liu et al. showed that Nrf2 expression was increased in intestinal tissue of pregnant rats after induction with ZEA and postulated that modulation of Nrf2 signalling pathway is a main response element in gut cells. This effect was simultaneous with formation of ROS and inflammatory status in cells (Liu et al. 2014). Based on Cheng and al. study along with the increasing concentration of ZEA the Keap1-Nrf2 pathway increases, which causes an upregulation in the expression of genes activated by the Nrf2 transcription factor. The similar results were observed in the ileum and mesenteric lymph nodes of post-weaning gilts (Cheng et al. 2019, 2020). Nrf2 activation in response to ZEA-induced oxidative stress was also observed in juvenile grass carp intestine; however, no changes in Keap1 levels were observed (Wang et al. 2019). The molecular effect of ZEA, mostly considered with its estrogenic properties in cells, is associated with induction od oxidative stress-induced apoptosis or autophagy and thus activation of Nrf2 is reported, especially in consideration of protective compounds which aim to reduce ZEA-induced oxidative stress. However, many research studies reporting ZEA-induced apoptosis did not consider Nrf2 signalling pathway as a molecular effect of ZEA, although oxidative stress induced by environmental factors is known to trigger different types of death in cells, e.g., necrosis, apoptosis and the role of Nrf2 seems to be crucial in these processes (Nam and Keum 2019).

### Ochratoxin A

*Aspergillus* and *Penicillium* fungi are mainly producing ochratoxins (OTs). These mycotoxins are found in various food products: cereals, dried fruit, meat, red wine, beer, nuts and coffee. The most dangerous member of this family is Ochratoxin A (OTA), classified as type 2B carcinogen, which has been found recently also in herbs used in traditional medicine (Veprikova et al. 2015; Chen et al. 2015; Su et al. 2018; Toman et al. 2018). OTA leads to many disorders both in humans and animals. After absorption, OTA is able to bind to albumin and in consequence, its half-life in the body is prolonged (Kőszegi and Poór 2016). Products of OTA metabolism are formed mainly via hydrolysis, hydroxylation, lactone opening and conjugation (Wu et al. 2011; Tao et al. 2018). Similarly to other mycotoxins, OTA is reported to enhance production of ROS and thus led to disturbance in cell cycle (Liu et al. 2012) and DNA, lipid and protein damage (Marin-Kuan et al. 2011). Its carcinogenicity is mainly associated with nephro- and hepato-toxicity (Marin-Kuan et al. 2011). It is proved that OTA affects expression of transcription factors involved in oxidative stress response. OTA reduces the expression of detoxifying enzymes via modulation of Nrf2 activity (Marin-Kuan et al. 2011). In the study of Boesh-Saadatamandi et al. OTA decreased expression of Nrf2 and HO-1 (and its downstream genes) in cells derived from pigs kidney and the authors postulated that downregulation of Nrf2 might be partially responsible for nephrotoxicity of OTA (Boesch-Saadatmandi et al. 2009). A similar effect was observed in porcine renal proximal tubular cells (Stachurska et al. 2013). In turn, Shanel et al. showed that OTA in human embryonic kidney cells (HEK293) induced expression of NF-κB (Raghubeer et al. 2017). Due to the fact that this factor is Nrf2 regulator, it seems that the expression of Nrf2 in these cells might be also affected. Interestingly, not only Nrf2 but its downstream target HO-1 might play a protective role in OTA toxicity: it was observed that OTA significantly affects murine kidneys. Furthermore, HO-1 knockout mice presented acceleration of OTA-induced fibrogenic, inflammatory and apoptotic effect (Loboda et al. 2017a). Similar effect was observed in mice lacking Nrf2 and interestingly, male mice were more sensitive to this effect than females, suggesting that the effect of its toxicity depends on gender (Loboda et al. 2017b). The decrease in Nrf2 activation induced by OTA was also observed in NRK-52E rat kidney cells were the influence of another oxidative stress regulator was suggested: hypoxia inducible factor 1α (HIF-1α) which activation was associated with angiogenesis (Liu et al. 2020). Induction of apoptosis and oxidative stress by OTA in chicken kidneys and liver was associated with modulation of Nrf2/Keap1 as well as PI3K/Akt signalling pathways as confirmed by Li et al. (2020a). The decreasing expression of Nrf2 and HO-1 was also observed after treatment of porcine renal proximal tubular cells with OTA (Stachurska et al. 2013). Shin et al. showed that OTA-induced oxidative stress in human hepatocytes is associated with aryl hydrocarbon receptor (AhR) as well as Nrf2, additionally the increase in the expression as well as nuclear localization of Nrf2 was observed (Shin et al. 2019). This fact suggests that phase II reactions are highly dependent on phase I detoxifying reactions in cell in response to OTA. OTA-induced liver injury was also observed in ducks, Zhai et al. indicated that observed effect might be modulated by antioxidants modulating intestinal microbiota such as tryptophan and glyceropholipid metabolism, significantly affected by OTA in ducks (Zhai et al. 2020). OTA induces oxidative stress and in consequence injury in murine heart muscle via Nrf2 signalling pathway: a decrease in heart weight, increased serum concentration of cardiac enzymes and antioxidant levels were observed (Cui et al. 2020). OTA, possibly due to its known carcinogenicity, in most of the studies decreases Nrf2 and its downstream targets in cells, whereas natural antioxidants are able to restore the OTA-induced decrease of Nrf2.

### Aflatoxin B1

More than 20 aflatoxins (AFs) are produced by *Asperegillus* species, mainly by *Aspergillus flavus* and *Aspergillus parasiticus*. AFs are slightly soluble in water and soluble in polar solvents. AFs presents acute toxicity at high doses and chronic toxicity at low doses (Ülger et al. 2020). AFs are absorbed in duodenum and transported to the liver- the most exposed organ to AFs toxicity, due to the first line of metabolism of these mycotoxins. However, AFs also affect others tissues such as kidney, pancreas or bladder (Benkerroum 2020). Six major AFs are distinguished: B1, B2, G1, G2, M1 and M2 from which Aflatoxin B1 (AFB1) is the most dangerous form, classified ascarcinogen 1 according to International Agency for Research on Cancer (IARC) (Ülger et al. 2020). AFB1 induces oxidative stress, leads to DNA damage and disturbance in mitochondrial permeability which was proved in tissues derived from different organisms (Theumer et al. 2010; Abdel-Aziem et al. 2011; Shi et al. 2015). As a carcinogen AFB1 induces DNA strand breaks, chromosomal abnormalities and mutation in p53 gene (Corcuera et al. 2015). As the result of AFB1 metabolism, Aflatoxin M1 may be formed, which is less carcinogenic than AFB1 (Wen et al. 2016). However, AFB1 may be metabolized by CYP450 to AFB1-exo-8,9-epoxide (AFBO)- a reactive form which binds to nucleic acids, proteins and thus lead to apoptosis, DNA-damage, disruption in signalling pathways and protein synthesis (Benkerroum 2020). One of the signalling pathways influenced by AFB1 seems to be Nrf2 pathway. Activation of Nrf2 was present in the most exposed organ to AFB1: liver. It was presented that AFB1 leads to decreased expression of Nrf2 and its downstream gene HO-1, simultaneously with oxidative stress and pro-inflammatory cytokines response in broiler chickens (Li et al. 2019b). Induction of Nrf2 signalling pathway was also observed in common carp (Kövesi et al. 2020b). The experiment conducted on human hepatocytes L02 cells revealed that caveolin-1 (CAV-1) interacts with Nrf2 in response to oxidative stress caused by AFB1 to trigger apoptosis in cells (Xu et al. 2020). A decrease in Nrf2 and HO-1 expression was also noticed in Wistar rats exposed to AFB1 and interestingly that effect was partially restored by administration of antidiabetic drug Sitagliptin (Ji et al. 2020). AFB1 affects lipid mitochondrial metabolism and oxidative stress response element Nrf2 after acute exposure in rats (Rotimi et al. 2019). Muhammad et al. suggested that AFB1 does not cause autophagy in broiler chicken livers, but induces inflammation and reduces expression of Nrf2 and HO-1, indicating that the switch between autophagy and inflammation might play a crucial role in AFB1 induced hepatotoxicity (Muhammad et al. 2018). Human hepatic HepG2 cells exposed to AFB1 presented an increased oxidative stress and hepatotoxicity with DNA damage and cytotoxicity with downregulation of Nrf2/HO-1 signalling pathway (Vipin et al. 2017). On the other hand, in primary broiler hepatocytes AFB1 caused upregulation of Nrf2 although the expression of its downstream genes decreased (Liu and Wang 2016). In broiler cardiomyocytes Wang et al. observed that AFB1 also upregulates expression of Nrf2 (Wang et al. 2017). It should be emphasized, that *Nrf2* knockout rats are more susceptible than wild type to toxic effect of AFB1 via decreased expression of its downstream genes (Taguchi et al. 2016). Based on the fact that AFB1 is a carcinogen, its effect on other tissues was also investigated. Zhou et al. observed that similarly to liver tissue, AFB1 induces cytotoxicity, oxidative stress and apoptosis in cow mammary epithelial cells associated with increase in the expression of Nrf2 signalling pathway components: Nrf2, Keap1, NQO1 and HO-1 after 3 hours of exposure (Zhou et al. 2019). The decreased expression of Nrf2 signalling pathway components was also observed in murine renal cells exposed to AFB1 (Yu et al. 2018a). Based on these studies Nrf2 seems to participate in carcinogenic effect of AFB1 and potentially the activators of Nrf2 signalling pathways both natural as well as chemical might serve as AFB1 detoxifying enzymes.

## Natural antioxidants

Due to fact that Nrf2 signalling pathway seems to participate in the toxic effect of mycotoxins on human and animal cells, searching of new antioxidative agents that in most cases activates Nrf2 expression, constitutes the aim of many research studies. In most cases, natural antioxidants are taken into consideration. Natural antioxidants are a group of chemical compounds extracted from plants which are commonly used in pharmacy as a supplements of diet or drugs used to prevent diseases. Many of the natural antioxidants are known to decrease oxidative stress induced by mycotoxins by restoring the expression of Nrf2 and its downstream targets (Table 2). The extract of *Scutellaria baicalensis*, known as baicalin, is a flavonoid which easily chelates with Zn and Cu to form a metal chelate complexes which possesses a higher antioxidant capabilities than flavonoid itself. Dietary supplemented baicalin zinc reduced DON-induced expression of p-Nrf2 and HO-1 in the piglets intestine (Zha et al. 2020a). Similar effect was observed for the complex of baicalin copper in another study (Zha et al. 2020b). Grape seed proanthocyanidin extract increased the expression of Nrf2 and its downstream genes decreased in the response to oxidative stress triggered by ZEA in murine liver and Sertoli cells (Long et al. 2016, 2017) as well as AFB1 induced oxidative stress and inflammation in broilers (Rajput et al. 2019). Curcumin seems to serve as Nrf2 activator in response to different mycotoxins. The increase in the expression of Nrf2 after curcumin exposure was present in ZEA- treated Leydig cells (Chen et al. 2020), AFB1- treated liver cells (Muhammad et al. 2018; Li et al. 2019b) and OTA- treated liver cells in ducks (Zhai et al. 2020). The toxic effect of AFB1 was also abolished by resveratrol in bovine mammary epithelial cell via modulation of Nrf2 (Zhou et al. 2019). Another natural compounds reported to increase the expression of Nrf2 in AFB1 treated cells are: lycopene (Yu et al. 2018a), ginger extract (Vipin et al. 2017) and coffee (Cavin et al. 2008). The phenolics rich extract of ginger in in vivo and in vitro study showed to limit hepatotoxic effect of AFB1. Moreover, it was proved that administration of extract from ginger led to increased expression of Nrf2/HO-1 pathway (Vipin et al. 2017). Coffee has an impact in the prevention against ROS produced in response to AFB1 via induction of Nrf2/ARE pathway (Cavin et al. 2008). Lycopene protected AFB1- induced renal injury in mice (Yu et al. 2018a). Similarly to AFB1, OTA-induced decrease in Nrf2 expression associated with oxidative stress might be also attenuated by natural antioxidants. Flavonoid luteolin regulates Nrf2 and HIF-1α signalling pathways to alleviate OTA-induced oxidative stress (Liu et al. 2020). Carotenoid astaxanthin similarly to luteolin protects against OTA-induced kidney injury (Li et al. 2020b), similarly to yeast selenium used as diet supplement (Li et al. 2020c). Also quercetin, a natural flavanol, stimulates translocation of Nrf2 to the nucleus and increases its expression after cell exposure to OTA and thus alleviate the toxicity of this mycotoxin (Ramyaa and Padma 2013; Ramyaa et al. 2014).

**Table 2:** Summary information about natural antioxidants affecting the expression of Nrf2 and its downstream genes after exposure to various mycotoxins

Mycotoxin	Natural ingredients	Influence on Nrf2	Influence on Nrf2 downstream genes	References
DON	Resveratrol	↑	↑GCLM, GCLC	Yang et al. (2019)
	Baicalin zinc	↓	↓HO-1	Zha et al. (2020a)
	Baicalin copper	↓	↓HO-1	Zha et al. (2020b)
ZEA	Grape-seed proanthocyanidin extract	↑	↑HO-1, NQO1, γ-GCS	Long et al. (2016)
	Curcumin	↑	↑HO-1↓Keap1	Chen et al. (2020)
AFB1	Curcumin	↑	↑HO-1	Zhang et al. (2016); Wang et al. (2018); Muhammad et al. (2018); Li et al. (2019b)
	Phenolics of ginger^a^	↑	↑HO-1	Vipin et al. (2017)
	Resveratrol	↑	↑HO-1, NQO1	Zhou et al. (2019)
	Grape-seed proanthocyanidin extract	↑	↑HO-1, GPx1, NQO1, GCLC	Rajput et al. (2019)
	Coffee	↑	↑HO-1, GCLC, NQO1	Cavin et al. (2008)
	Lycopene	↑	↑ NQO1, GCLM, GCLC	Yu et al. (2018a)
OTA	Quercetin^b^	↑	–	Ramyaa and Padma (2013); Ramyaa et al. (2014)
	Luteolin	↑	↑ HO-1, γ-GCS and Gpx-1	Liu et al. (2020)
	Astaxanthin	↑	↓ Keap-1, ↑HO-1	Cui et al. (2020)
	Yeast selenium (Se-Y)	↑	↑HO-1, GSH-px, GLRX2	Li et al. (2020a, c)
	Curcumin	↑	↑HMOX1	Zhai et al. (2020)

↓ downregulated, ↑ upregulated

^a^In human cells

^b^In human and animal cells

## Conclusions

It is generally known that mycotoxins affect human and animal cells by disruption of homeostasis and consequently generation of oxidative stress. It is also known that we are not able to eliminate mycotoxins contamination in food, but understanding its molecular effect on human and animal health might help to search for potential detoxifying agents. Studies from last years showed that Nrf2 transcription factor might play a crucial role in the cellular defence mechanisms. This review confirmed that the most common and well known mycotoxins affect expression of Nrf2 factor and thus lead to decreased expression of its downstream genes which are directly involved in the detoxification of the organism. Interestingly, this literature survey also suggested that the effect of mycotoxins on Nrf2 expression is more complex and involved different singling pathways: Nrf2/ARE, HIF-1α, PI3K/Akt, Ahr etc. Nevertheless, in most studies Nrf2 expression and its localization is affected and thus seems to appear as a good target for potential drugs and/or antioxidants. In most of the cases mycotoxins caused decreased expression of Nrf2, however, it should be emphasized that Nrf2 might play a dual role in cells and thus more research studies should be carried out in the future to both evaluate Nrf2 role in mycotoxins detoxication as well as its potential activators serving as mycotoxins detoxifying agents.
